# Study of The Gas-Swelling Mechanisms in Silicon Carbide Ceramics under High-Temperature Irradiation with Helium Ions

**DOI:** 10.3390/ma16175750

**Published:** 2023-08-22

**Authors:** Kymbat M. Tynyshbayeva, Artem L. Kozlovskiy, Ruslan V. Rakhimov, Vladimir V. Uglov, Maxim V. Zdorovets

**Affiliations:** 1 Engineering Profile Laboratory, L.N. Gumilyov Eurasian National University, Astana 010008, Kazakhstan; tinishbaeva.kimbat@mail.ru (K.M.T.);; 2 Laboratory of Solid State Physics, The Institute of Nuclear Physics, Almaty 050032, Kazakhstan; 3 Department of Solid State Physics, Belarusian State University, 220050 Minsk, Belarus

**Keywords:** SiC ceramic, gas-filled bubbles, swelling, thermal conductivity coefficient, strength characteristics

## Abstract

The purpose of this work is to simulate the processes of gaseous swelling in SiC ceramics as well as the associated changes in strength and thermophysical properties under high-temperature irradiation with helium ions. The choices of irradiation conditions (irradiation temperatures of 700 and 1000 K) and irradiation fluences (10^15^–10^18^ ion/cm^2^) are based on the possibilities of modeling the processes of destructive changes in the near-surface layer as a result of the accumulation of gas-filled inclusions during high-dose irradiation. During this study, it was found that an increase in the irradiation temperature of the samples from 700 to 1000 K leads to a decrease in the resistance to gas swelling, since with the temperature increase, the mobility of implanted helium in the near-surface layer grows, which results in an increase in the size of gas-filled bubbles and, as a result, accelerated destruction of the damaged layer. It has been established that in the case of irradiation at 700 K, the critical fluence for swelling associated with the formation of visible gas-filled bubbles on the surface is 5 × 10^17^ ion/cm^2^, while for samples irradiated at a temperature of 1000 K, the formation of gas-filled bubbles is observed at a fluence of 10^17^ ion/cm^2^. Measurements of the thermal conductivity coefficient showed that the formation of gas-filled bubbles leads to a sharp deterioration in heat transfer processes, which indicates that the created defective inclusions prevent phonon heat transfer. Changes in the strength characteristics showed that a decrease in hardness occurs throughout the entire depth of the damaged ceramic layer. However, with a rise in the irradiation fluence above 10^17^ ion/cm^2^, a slight damaged layer thickness growth associated with diffusion processes of helium implantation into the near-surface layer is observed. The relevance of this study consists in obtaining new data on the stability of the strength and thermophysical parameters of SiC ceramics in the case of helium accumulation and its subsequent radiation-induced evolution in the case of irradiation at temperatures of 700 and 1000 K. The data obtained during the experimental work on changes in the properties of ceramics will make it possible to determine the potential limits of their applicability in the case of operation under extreme conditions at elevated temperatures in the future.

## 1. Introduction

The problem of gas swelling during the accumulation of helium or hydrogen in the near-surface layer of structural materials of nuclear reactors—in particular, the materials of the first wall or materials of the assembly of fuel elements that hold nuclear fuel in the core—is very important not only from a practical point of view but also a fundamental one [1,2,3,4,5]. Moreover, while in the case of stainless steels and alloys, this problem has already been studied quite well over the past 50–70 years, for ceramics, the problem associated with a destructive change in properties as a result of the accumulation of helium and hydrogen, leading to the formation of gas-filled bubbles, has not been sufficiently studied. It should also be noted that the transition to the operation of high-temperature nuclear reactors, which is accompanied by the transition to new types of structural materials including ceramics (carbides, nitrides, oxides) [5,6,7,8] alongside high-entropy alloys, requires a detailed study of the problem of the possible accumulation of helium in the near-surface layer as well as the consequences associated with its accumulation [9,10].

In the case of an increase in the operating temperature of the core, which makes it possible to achieve a higher degree of nuclear fuel burnup, the concentration of the resulting implanted helium increases, and its mobility in the damaged layer also increases [11,12]. An increase in the mobility of implanted helium as well as its low solubility can lead to an acceleration of swelling processes due to the formation of many gas-filled bubbles. Similar effects have been observed in steels, but this issue has not been sufficiently studied in ceramics. An increase in helium mobility in the surface layer at elevated temperatures, together with an increase in thermal vibrations of atoms in the crystal lattice, can lead to an increase in the degree of distortion and deformation in the crystal structure [13,14]. A change in the concentration of deformation inclusions in the damaged layer can have a negative impact on both the strength properties and the mechanisms of heat transfer. In turn, the deterioration of heat transfer can lead not only to the appearance of local areas of overheating but also to a decrease in heat removal from the core or fuel element, which will lead to the destabilization of the reactor [15]. A decrease in strength properties as a result of the formation of gas-filled bubbles in the near-surface layer can lead to partial peeling of the surface as a result of the bubbles’ detonative opening (by rupturing) and can also lead to the possible formation of microcracks and a decrease in the fracture toughness of ceramics [16,17]. 

The purpose of this study, considering all the above, is to study the effect of irradiation temperature on the processes of gas swelling in SiC ceramics under irradiation with He^2+^ ions as well as to evaluate their effect on the change in the strength and thermophysical parameters of ceramics. The choice of SiC ceramics as objects for research is due to their great potential for use, along with other types of ceramics (AlN, Si_3_N_4_, ZrN, ZrO_2_, BeO, MgO), as structural materials for high-temperature nuclear reactors as well as in ceramic containers for storing spent nuclear fuel [18,19,20,21,22]. The simulation results of high-temperature irradiation with He^2+^ ions will make it possible to determine the critical fluences at which swelling of ceramics occurs as well as to establish the relationship between structural distortions caused by swelling and changes in the strength and thermophysical properties of ceramics. As the main methods for the assessment of the concentration effect and dose dependences of helium accumulation in a damaged near-surface layer on the change in thermophysical and strength properties, methods of determining the heat flux [23] and the hardness on the surface and in depth along the trajectory of incident ions were used. Visualization of structural and morphological changes was performed using scanning electron microscopy methods. The use of the combination of these analysis methods, as well as the prospect of their use for assessment of the radiation damage kinetics in carbide ceramics, was employed in a number of works [24,25,26], in which unique data on the properties of these types of ceramics were obtained using these methods.

## 2. Experimental Section

Polycrystalline ceramics of silicon carbide (SiC) with a hexagonal type of crystal lattice, spatial syngony *P3m1(156)*, were chosen as samples for research. The original samples had a density of 3.2 g/cm^3^ and were purchased from Dongguan Mingrui Ceramic Tech. Co., Ltd. (Dongguan city, Guangdong, China). Before the experiments, the samples were subjected to grinding and polishing in order to create a smooth surface, the roughness of which was no more than 5 nm. The samples were 5 µm thick pellets with a diameter of 10 mm. 

Irradiation of the samples, in order to simulate the processes of gaseous swelling during the accumulation of implanted helium, was performed on the DC-60 accelerator located at the Institute of Nuclear Physics of the Ministry of Energy of the Republic of Kazakhstan (Almaty, Kazakhstan). The samples were irradiated in a vacuum on a special ceramic holder–heater that makes it possible to maintain a given sample temperature during the entire irradiation time in a given range with an error of ±5–10 K, which is no more than 1% for high irradiation temperatures. The samples were heated to the predetermined temperatures of 700 K and 1000 K at a rate of 10 K/s, and the heating rate was controlled using thermocouples placed on the opposite side of the heater from the sample. The control over the set irradiation fluence was carried out using Faraday cups as well as calculated parameters based on the values of the ion beam current density. During irradiation, the current density was maintained at the same level—150 ± 2 nA/s. The irradiation fluence of the samples under thermal irradiation was 10^15^–10^18^ ion/cm^2^. The irradiation fluence was chosen with the possibility of modeling the processes of accumulation of implanted helium in a near-surface layer with a thickness of no more than 500 nm with a concentration of up to 5 at. %, as well as establishing critical values at which gas-filled bubbles are formed on the surface of ceramics, indicating swelling. As is known from the a priori literature data, swelling processes under similar irradiation conditions (He^2+^ ion energy up to 100 keV) are initiated at fluences above 10^17^ ion/cm^2^.

The morphological features resulting from gaseous swelling were studied using the scanning electron microscopy method implemented using a Jeol 7500F microscope (Jeol, Tokyo, Japan). The resulting images reflect the dynamics of changes in the size and number of gas-filled bubbles formed on the ceramic surface depending on the irradiation fluence. 

The determination of the change in strength characteristics was made using the methods of indentation, single compression and determination of the coefficient of dry friction. For indentation, a Duroline M1 microhardness tester (Metkon Instruments Inc., Riga, Latvia) and a TI Premier nanoindenter (Bruker, Berlin, Germany) were used. Indentation was carried out at an indenter load of 10 mN. The measurement was carried out in two modes. The first mode included the measurement of hardness on the surface of the samples in order to determine the hardness and crack resistance as well as to establish the softening effect depending on the irradiation fluence. The second mode of measurements was carried out on side cleavages along the path of ions in the material to determine the uniformity of changes in the strength properties of ceramics over depth. A TI Premier nanoindenter was used for measurements, and a Berkovich pyramid was used as an indenter. The depth measurement step was 50 nm. 

Determination of the softening effect was carried out by comparative analysis of changes in the hardness values of the samples before and after irradiation, and the estimate of the softening value was calculated as a percentage. Based on the evaluation of indenter prints, the size and shape of microcracks and the crack resistance coefficient were determined. 

The measurement of resistance to cracking and crack formation, as well as the ability to withstand the maximum load in a single compression, was carried out using an LFM-L 10kH testing machine (Walter + Bai AG, Löhningen, Switzerland). The compression rate was 5 mm/min. Based on the data obtained, the values of the maximum pressure the ceramics were capable of withstanding under a compressive load were determined. 

Tests for measuring the coefficient of dry friction were performed using a UNITEST 750 tribometer (Ducom Instruments, Bengaluru, India). The tests were carried out by successive tests with a ball-shaped indenter, which acted on the surface under a load of 100 N. The number of friction repetitions was 20,000. 

Tests for thermal conductivity and determination of the thermal conductivity coefficient were carried out according to the method for determining the longitudinal heat flux in the range of measured temperatures from 20 to 800 °C. Measurements were carried out using thermocouples placed on both sides of the sample, followed by heating the sample on one side and measuring the temperature of the sample on the other side. For measurements, a KIT-800 device (Teplofon, Moscow, Russia) was used. 

## 3. Results and Discussion

As is known, the mechanisms of swelling or formation of gas-filled bubbles are associated with the agglomeration of implanted helium in the near-surface layer in cavities formed by the structural distortion of the crystal lattice associated with the interaction of incident ions with the near-surface layer as well as ionization processes. At the same time, these swelling effects can be most clearly observed using visualization methods using scanning electron microscopy of the sample surface, depending on the irradiation fluence.

**Figure 1:**
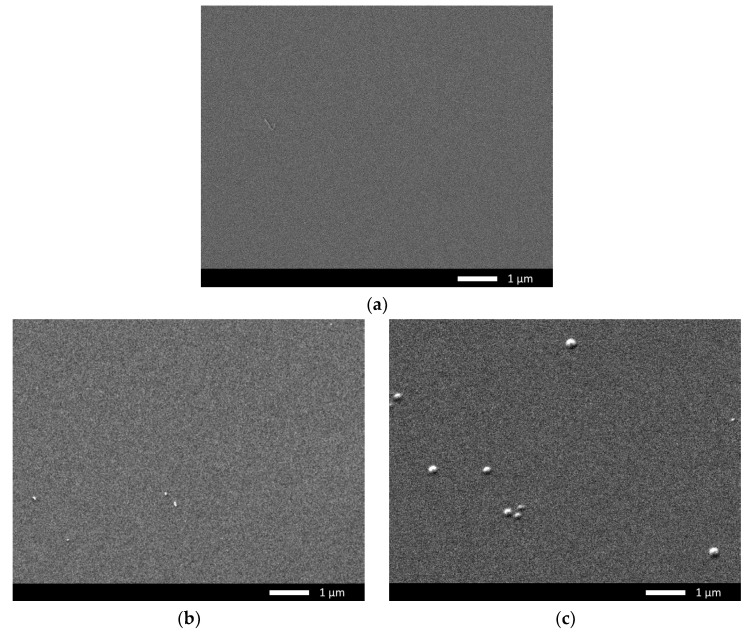

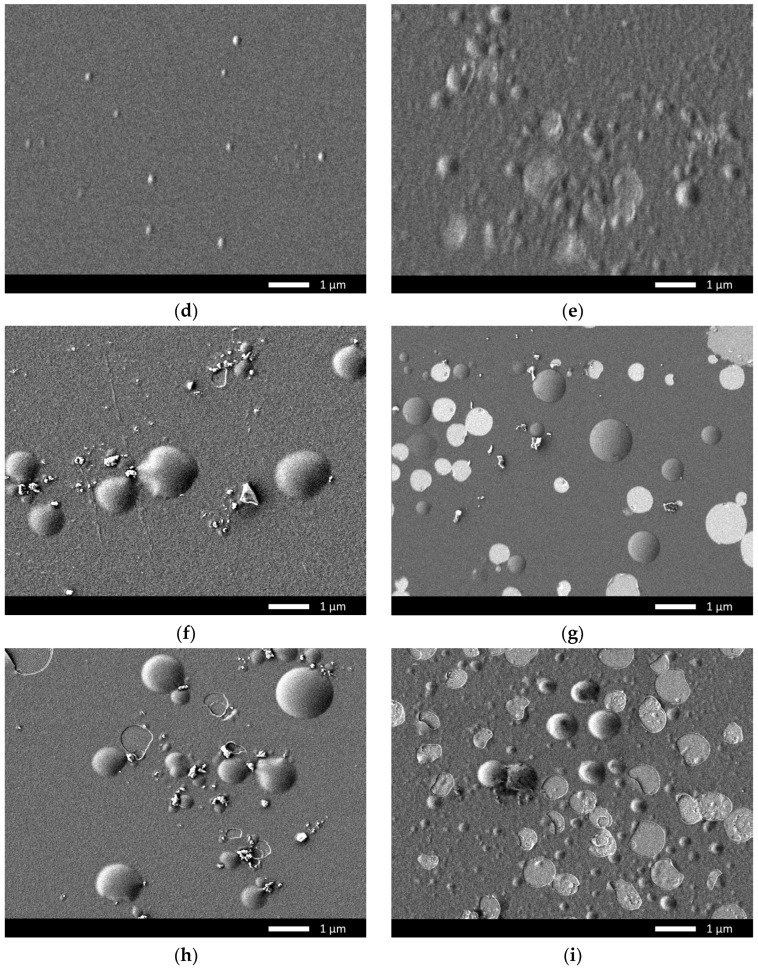
SEM images of the surface of ceramics exposed to irradiation with He^2+^ ions at various temperatures (SEM images taken in LEI mode at 15,000× magnification): (**a**) pristine; (**b**) irradiated to 10^17^ ion/cm^2^ and temperature 700 K; (**c**) irradiated to 10^17^ ion/cm^2^ and temperature 100 K; (**d**) irradiated to 5 × 10^17^ ion/cm^2^ and temperature 700 K; (**e**) irradiated to 5 × 10^17^ ion/cm^2^ and temperature 1000 K; (**f**) irradiated to 7 × 10^17^ ion/cm^2^ and temperature 700 K; (**g**) irradiated to 7 × 10^17^ ion/cm^2^ and temperature 1000 K; (**h**) irradiated to 10^18^ ion/cm^2^ and temperature 700 K; and (**i**) irradiated to 10^18^ ion/cm^2^ and temperature 1000 K.

Figure 1a–e show SEM images of the evolution of the nucleation and subsequent growth of gas-filled bubbles in the near-surface layer of ceramics depending on the increase in the irradiation fluence, which leads to an increase in the concentration of implanted helium. As can be seen from the presented SEM images of samples exposed to irradiation with a fluence of 10^17^ ion/cm^2^ (see the data in Figure 1b), in the case of irradiation at a temperature of 700 K, there are no obvious structural changes associated with the formation of bubbles or hillocks on the surface. At the same time, for samples irradiated at a temperature of 1000 K, the appearance of single spherical inclusions, 150–200 nm in diameter and characteristic of the nucleation of gas-filled bubbles, is observed on the surface. The appearance of such inclusions indicates helium agglomeration in structural cavities, followed by their deformation and extrusion to the surface, which is in good agreement with several works [27,28]. The small size and the distance from each other of the formed bubbles indicate a sufficiently high resistance of ceramics to the formation of such inclusions at given irradiation fluences. At the same time, the absence of such inclusions in samples irradiated at a temperature of 700 K and the same fluence indicates that agglomeration into gas-filled bubbles has a pronounced temperature dependence associated with a more accelerated helium mobility at high temperatures. An increase in the irradiation fluence up to 5×10^17^ ion/cm^2^ in the case of ceramics irradiated at 1000 K leads to an increase in both the number of bubbles formed and their size in diameter. It should be noted that the formation of bubbles does not occur everywhere over the entire surface of the sample but in the form of agglomerations. This formation can be explained by the fact that helium accumulation in the damaged layer occurs in areas with structural distortions or pores in which helium accumulates, with a subsequent increase in the concentration of structurally distorted areas near the agglomeration center. In this regard, the formation of gas-filled bubbles mainly occurs in the vicinity of these areas, which leads to their merging into larger bubbles. In the case of samples irradiated at a temperature of 700 K with a fluence of 5 × 10^17^ ion/cm^2^, the appearance of single bubbles is observed, the size of which does not exceed 100 nm. This indicates that the swelling processes associated with helium agglomeration are less intense than when the samples are irradiated at a temperature of 1000 K. Such a difference in the dependence of the formation of gas-filled bubbles on the surface of the damaged layer on the irradiation fluence and temperature can be explained by the effects associated with an increased rate of helium agglomeration at elevated temperatures, leading to the association of small bubbles formed in the damaged layer into larger ones, which leads to their displacement to the surface as well as the formation of spherical bulges on the surface. Similar effects of agglomeration at elevated temperatures, in particular during post-radiation annealing to visualize gas-filled bubbles, were observed in steels and alloys [29,30,31,32]. In the case of a further increase in the irradiation fluence, for both temperatures, the formation of gas-filled bubbles of a larger shape is observed (the average size of large bubbles exceeds 1 μm in diameter), which indicates migration and agglomeration processes of implanted helium in the damaged near-surface layer. At the same time, in the case of an irradiation temperature of 1000 K, craterlike inclusions are observed on the surface, indicating an explosive mechanism for the opening of bubbles as a result of the accumulation of excess pressure in them, leading to the destruction of the bubble. It is also worth noting the fact that, according to the presented SEM images, the opening of bubbles occurs most intensively in the area of their agglomeration on the surface. Consequently, the most pronounced effect of explosive opening is manifested in the case when there is not only excess pressure inside the bubble, which is explained in sufficient detail in the Evans model [33,34], but also external mechanical action from the nearest bubbles. In the case of samples irradiated at a temperature of 700 K, this effect of explosive opening of bubbles is not observed, and the main effect of swelling is associated with the formation of large single bubbles with a diameter of more than 1 μm, near which smaller bubbles are formed. This effect is similar to the observed effect of the swelling of samples irradiated at a temperature of 1000 K at a lower fluence. This is one of the confirming facts of the effect of irradiation temperature on the rate of helium migration and agglomeration in the near-surface damaged layer as well as the subsequent evolution of bubbles associated with their explosive opening and degradation of the ceramic surface.

At the maximum irradiation fluence of 10^18^ ion/cm^2^, in the case of samples irradiated at a temperature of 700 K, we observe not only the formation of large gas-filled bubbles and agglomerates of smaller bubbles around them but also a partial opening of the bubbles with the formation of craters. At the same time, some of the craters are located near other bubbles, which is also a confirming fact of the possible influence of external pressure formed near the bubbles on the acceleration of the opening of neighboring bubbles. Also, flakes near the craters are observed on the surface of the sample, indicating an explosive mechanism for the opening of bubbles followed by exfoliation of the damaged layer. 

In the case of samples irradiated at a temperature of 1000 K, the effect of the formation of the second generation is observed inside the formed craters as well as near them. The formation of smaller bubbles inside the craters indicates that helium agglomeration also occurs in deeper layers, which can lead to the propagation of structural deformation distortions associated with helium agglomeration, which can significantly affect the change in strength properties. Also, the formation of roughness is observed on the surface of the irradiated samples, the presence of which can lead to an increase in friction. 

**Figure 2:**
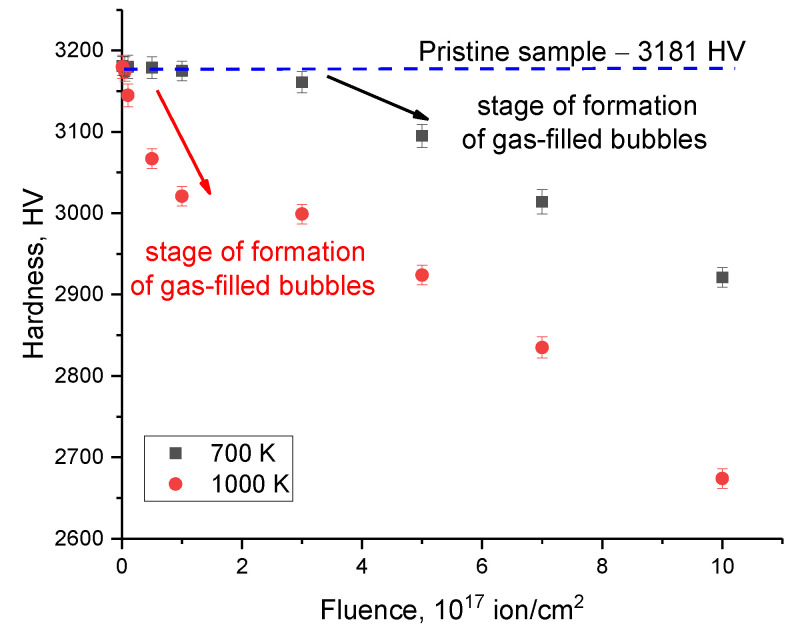
Results of evaluation of changes in hardness values of the near-surface layer of ceramics in cases of irradiation at different temperatures (measurements are taken from the surface of samples; blue dotted lines indicate hardness value for the initial sample and arrows (red and black) reflect hardness reduction trends).

Figure 2 shows the results of measuring the hardness of ceramics on the surface of samples subjected to irradiation at different temperatures. The general view of the presented changes indicates the influence of the irradiation temperature on the changes in hardness values with an increase in the irradiation fluence, and, as a result, the accumulation of radiation damage and implanted helium in the near-surface layer. As can be seen from the data presented, for ceramic samples irradiated at a temperature of 700 K at fluences less than 3 × 10^17^ ion/cm^2^, no change in hardness is observed, which indicates a sufficiently high resistance of ceramics to embrittlement and softening. At the same time, the main changes in hardness associated with its decrease are observed when the irradiation fluence reaches more than 3 × 10^17^ ion/cm^2^, while the trend of decreasing hardness values is close to linear depending on the irradiation fluence. It should also be noted that the most pronounced changes in hardness are observed in the area of fluences characteristic of the formation of gas-filled bubbles on the surface of the samples (5 × 10^17^–10^18^ ion/cm^2^). In this case, the increase in fluence, which leads to the accumulation of the concentration of implanted helium in the near-surface layer, is accompanied by the softening of the damaged layer, and the softening itself is due to the effects of deformation distortions associated with helium migration and its agglomeration. In the case of irradiation at a temperature of 1000 K, a decrease in hardness is observed at a fluence above 10^17^ ion/cm^2^, which indicates that the degradation of the damaged layer with an increase in the irradiation temperature proceeds more intensively than at an irradiation temperature of 700 K, and the softening processes themselves are more pronounced. It should also be noted that the change in hardness in the case of irradiation at a temperature of 1000 K has a significantly different trend in the region of 10^17^–5 × 10^17^ ion/cm^2^, characterized by the presence of an insignificant plateau, the presence of which may be due to the effect of accumulation of structural distortions during helium agglomeration and the subsequent increase in the volume of gas-filled bubbles, which is clearly observed in the presented SEM images (see Figure 1b,c). In this case, the so-called effect of strain hardening is possible, associated with the creation of additional obstacles for the mechanical action of the indenter, which leads to a decrease in the hardness decrease trend. In the case when, for samples irradiated at a temperature of 1000 K, a partial opening of gas-filled bubbles is observed, a sharp decrease in hardness occurs of more than 15–20% compared to the initial value. It should also be noted that for both batches of samples irradiated at temperatures of 700 K and 1000 K, an increase in the irradiation fluence above 5×10^17^ ion/cm^2^ leads to the same decrease in the trend of hardness values while maintaining a difference in values of approximately 10–12%. Such a difference in the hardness values may be due to the fact that, with an increase in the irradiation temperature from 700 K to 1000 K, the processes of degradation of the damaged layer occur more intensively, which is clearly seen in the SEM image data (the process of swelling and opening of the bubbles occurs more intensively for samples irradiated at a temperature of 1000 K). 

**Figure 3:**
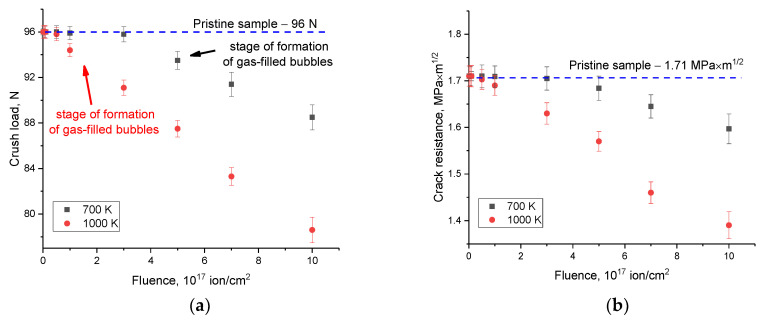
Strength test results: (**a**) tests for single compression of samples at a constant speed; and (**b**) tests to determine the crack resistance of specimens.

An important factor determining the resistance of ceramics to external influences is the preservation of their resistance to cracking under mechanical influences (external pressure) on the damaged layer. Figure 3 shows the results of estimating the change in the value of the maximum load that the ceramic can withstand under a single compression at a constant rate, as well as the value of crack resistance, calculated on the basis of changes in strength properties. These values reflect the resistance of ceramics to fracture under external pressures and crack formation. As can be seen from the data presented, in the initial state, ceramics are able to withstand a pressure of more than 95 N, which is a fairly high value of resistance to external influences. At the same time, this value remains within the measurement error range for irradiated samples at an irradiation temperature of 700 K in the range of irradiation fluences up to 3 × 10^17^ ion/cm^2^, and for samples irradiated at a temperature of 1000 K with irradiation fluences of 10^15^–10^16^ ion/cm^2^. At the same time, the trends in the decrease in stability are similar to changes in the hardness values of ceramic samples depending on the irradiation fluence, which indicates that the changes in the mechanical properties of ceramics (hardness, strength and resistance to cracking and fracture) have a similar character, and as a result, the mechanisms that lead to changes in mechanical properties have an equivalent effect on their changes.

**Figure 4:**
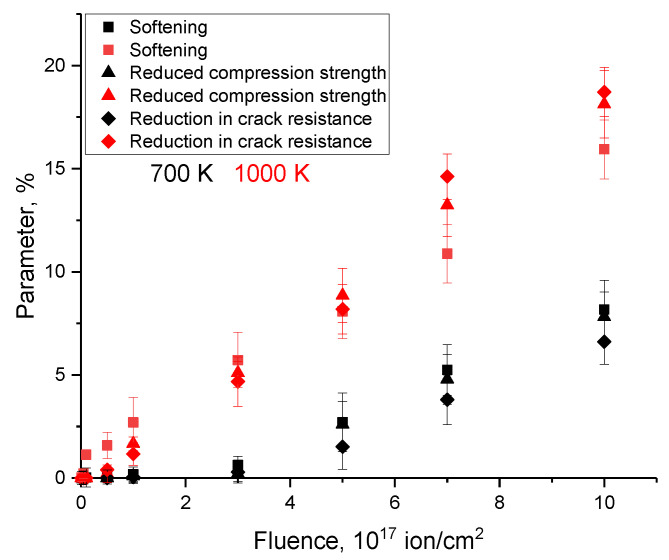
Results of a comparative analysis of trends in changes in the mechanical properties of ceramics as a result of irradiation.

Trends in mechanical properties (softening, decrease in resistance to external influences, decrease in crack resistance), reflecting the degradation of the damaged layer as a result of irradiation, are shown in Figure 4 depending on the irradiation temperature. As can be seen from the data presented, the changes in the values above have a similar form depending on the irradiation fluence, with the difference that the most pronounced changes in the mechanical characteristics for samples irradiated at a temperature of 700 K are observed from an irradiation fluence of 3–5 × 10^17^ ion/cm^2^, while a decrease in mechanical characteristics for samples irradiated at a temperature of 1000 K is observed from a fluence of 10^17^ ion/cm^2^. At the same time, an increase in the irradiation temperature from 700 K to 1000 K at irradiation fluences above 3 × 10^17^ ion/cm^2^ leads to an increase in the degradation of strength parameters by more than 5–7% while maintaining this trend even at high irradiation fluences. Such a difference in the change in strength properties can be explained by the effects of accelerated migration and degradation of the near-surface damaged layer with an increase in the irradiation temperature. And the increase in the difference in the degradation of strength properties with increasing irradiation fluence can be explained by a more intense accumulation of structural deformation inclusions in the damaged layer during the detonative opening of gas-filled bubbles. 

One of the important parameters in determining the strength properties is the assessment of changes in the hardness of the samples along the ion trajectory in the material. This can occur at a much greater depth than the maximum ion path because the ionization processes of interaction of ions with the structure can be different in depth, as can the structural distortions and deformations caused by them, which leads to softening. This is primarily due to the effects of defect migration, as well as the formation of primary knocked-on atoms, changes in the distribution of electron density associated with the processes of interaction of incident ions with electron shells and the effects of implantation and subsequent migration of helium in the structure. The formation of primary knocked-on atoms along the trajectory of ions in the damaged layer leads to the accumulation of deformation distortions of the crystal structure due to the migration processes of primary knocked-on atoms and their interaction with the crystal lattice. In the case of an increase in the irradiation fluence, the accumulation of deformation distortions leads to structural disordering of the damaged layer and a subsequent deterioration in the strength and thermophysical parameters of the ceramics.

**Figure 5:**
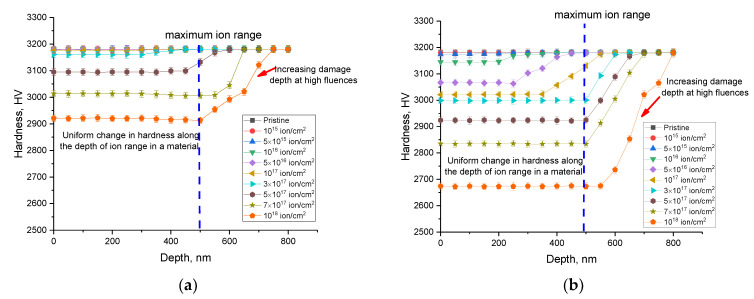
The change in hardness along the trajectory of ion movement in ceramics, determined by evaluating the hardness values performed by the method of indentation on a side cleavage: (**a**) at an irradiation temperature of 700 K; and (**b**) at an irradiation temperature of 1000 K.

Figure 5a,b reveal the assessment results of changes in the hardness values of ceramics irradiated at different temperatures, performed by measuring the hardness values along the ion trajectory on the side cleavage. These results reflect the changes in hardness with depth, according to which, at high irradiation fluences (above 3–5 × 10^17^ ion/cm^2^), a decrease in hardness occurs with equal probability along the entire ion trajectory in the material, and structural distortions caused by irradiation are equally probable along the entire ion trajectory. This effect is due to the fact that the most pronounced changes in hardness are due to structural and deformation distortions caused by the interaction of incident ions with the crystal structure and ionization processes as a result of the interaction of ions with the electronic subsystem. As shown in [35], the occurrence of mechanical residual stresses along the trajectory of ions in the material is associated with ionization electronic losses of ions, which dominate along the entire trajectory of motion over nuclear losses. In this case, the accumulation of structural distortions caused by the accumulation of mechanical residual stresses occurs almost uniformly along the entire ion trajectory. The observed experimental results of the change in hardness with depth are in good agreement with the results of [35], and the proposed description of the dependence of the formation of residual mechanical stresses and structural distortions can be used to explain the observed changes in hardness with depth.

In the case of low irradiation fluences (below 3 × 10^17^ ion/cm^2^), the change in strength properties along the ion motion trajectory in the material is either not observed or has a much smaller value. This effect manifests itself most clearly when samples are irradiated at a temperature of 1000 K, for which a change in the profile of hardness degradation with depth depending on the irradiation fluence is clearly seen. In the case of high irradiation fluences, a decrease in hardness is observed, which exceeds the maximum ion path depth in the material (more than 500 nm), which can be explained by several factors.

**Figure 6:**
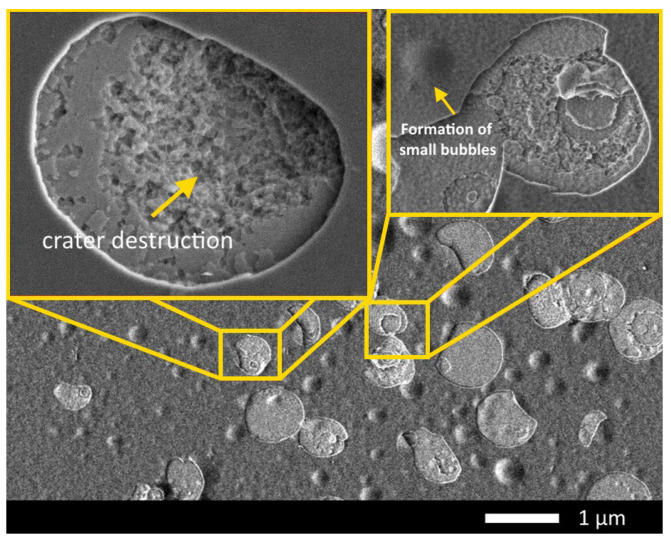
Example of the surface destruction of SiC ceramics in the case of irradiation at a temperature of 1000 K and a fluence of 10^18^ ion/cm^2^. (Areas with characteristic structural changes in the form of exploded gas-filled bubbles on the ceramic surface are highlighted in yellow).

The observed changes in hardness along the ion path profile in ceramics at high irradiation fluences can be explained by the effect of implanted helium migration not only on the surface but also deep in the material. Also, such changes can be associated with the effect of the detonative opening of gas-filled bubbles (the formation of craters), the formation of which is accompanied by the accumulation of deformation distortions and stresses near the formed cavities. In this case, an increase in the growth of bubbles due to the coalescence of helium bubbles in the cavities can exert mechanical stress not only on the surface but also deep in the material, as was shown in the work [36]. According to the calculations by Hofmann, F., et al., [36] the greatest structural distortion of the crystal lattice occurs perpendicular to the surface, which in the case of large bubbles can lead to a strong structural distortion due to deformation stresses. Figure 6 shows examples of crater destruction of the surface as well as the nucleation of bubbles near destroyed craters on the surface of SiC ceramics in the case of irradiation at a temperature of 1000 K and a fluence of 10^18^ ion/cm^2^. At the same time, as can be seen from the presented SEM images, the craters formed after the opening of the bubbles have a sufficiently large depth (more than several hundred nanometers), while the destruction has a clearly conical shape in depth, which indicates that swelling occurs not only towards the surface by squeezing the bubble outward but also deep into the damaged layer. Such observed changes indicate that the gas-dynamic model proposed by Evans for the expansion of a cavity with accumulated helium during its agglomeration [33,34] is accompanied not only by upward swelling but also by the occurrence of deformation distortions deep into the damaged layer. In the case of high irradiation fluences or high concentrations of implanted He^2+^ ions, this can lead to destabilization of the structure to a much greater depth than the maximum ion path length in the material.

**Figure 7:**
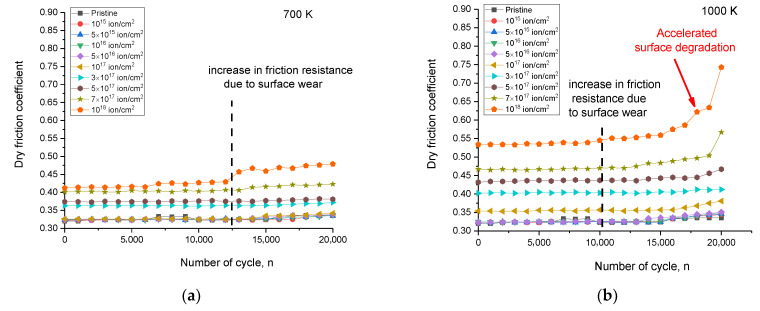
The results of changes in the dry friction coefficient of the surface of ceramics subjected to irradiation at different temperatures: (**a**) 700 K; and (**b**) 1000 K.

Figure 7 shows the results of changing the value of the coefficient of dry friction in the course-of-life tests to determine the resistance to external mechanical influences (friction) during a long exposure (20,000 cycles). These results reflect not only changes in surface degradation of irradiated samples during long-term testing but also changes in surface roughness as a result of irradiation, leading to the friction coefficient growth.

As can be seen from the presented data, the most pronounced changes in the friction coefficient (in the direction of increase), indicating surface degradation, are manifested for ceramic samples irradiated at a temperature of 1000 K. These changes are associated not only with an increase in the average value of the friction coefficient with an increase in the irradiation fluence, which indicates surface degradation due to the formation of gas-filled bubbles and crater inclusions, but also with accelerated surface degradation during long-term tests. Such degradation is primarily due to the fact that at high irradiation fluences, structural distortions associated with the coalescence of helium bubbles and their subsequent growth lead to degradation and wear of the damaged layer, accompanied by surface exfoliation. The friction coefficient growth at low irradiation fluences is due to the effects of roughness; these effects lead to the creation of additional obstacles during friction tests. In the case of samples irradiated at a temperature of 700 K, the changes in the dry friction coefficient are insignificant, and the most pronounced changes are observed at maximum irradiation fluences.

**Figure 8:**
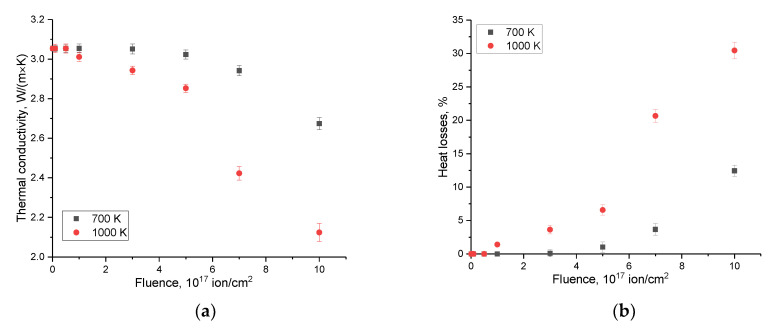
The results of measurements of changes in thermophysical parameters: (**a**) thermal conductivity coefficient; and (**b**) heat losses.

Figure 8 shows the results of changes in the thermophysical parameters (thermal conductivity and heat losses) of the studied SiC ceramics depending on the fluence and irradiation temperature. The change in these values reflects the deterioration of the thermophysical parameters of ceramics exposed to ionizing radiation.

The most pronounced changes in thermophysical parameters associated with the deterioration of thermal conductivity and an increase in heat losses are observed at fluences above 3–5 × 10^17^ ion/cm^2^, for which, as shown above, a decrease in strength characteristics is observed associated with the formation of gas-filled bubbles in the damaged layer. In this case, the formation of gas-filled bubbles is accompanied by an increase in deformation distortions of the structure, and these distortions in turn lead to the creation of additional scattering centers in the phonon heat transfer mechanism. Thus, we can conclude that there is a direct connection between the change in strength characteristics and the decrease in heat-conducting properties. 

**Figure 9:**
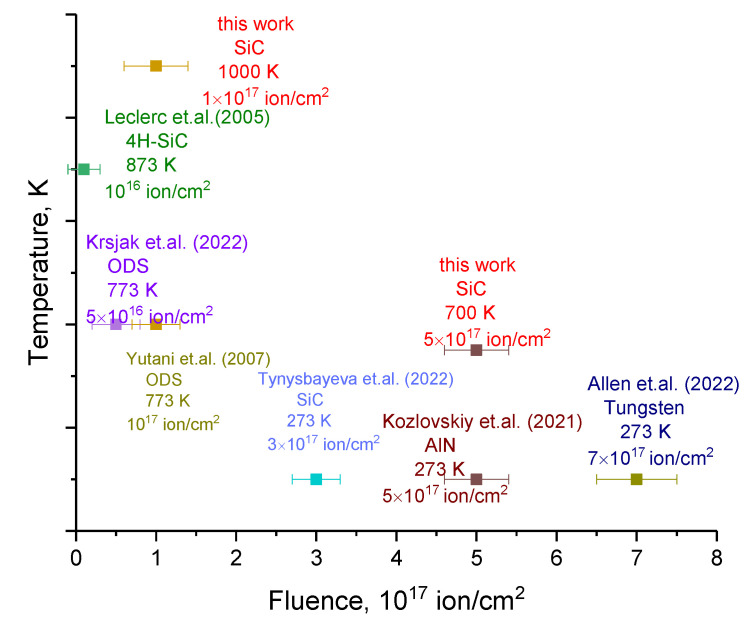
Comparative data on the effects of swelling in materials depending on the fluence of irradiation [37,38,39,40,41,42].

Figure 9 shows comparative data for various materials (ODS steels, tungsten, AlN ceramics and 4H-SiC single crystals) in which swelling effects were observed at different irradiation temperatures. These data make it possible to estimate the convergence of the swelling effects observed in this work in the studied SiC ceramics with other materials. 

The results obtained have a very good correlation with the literature data, which indicates that this type of ceramics can be used in structural materials capable of operating at high temperatures. So, for example, the observed swelling and formation of gas-filled bubbles in ODS steels [37,38] at high temperatures (773 K) occur at fluences much lower (10^16^–10^17^ ion/cm^2^) than similar effects observed in SiC [39] ceramics irradiated at a temperature of 700 K. At the same time, the observed swelling in SiC ceramics at a fluence above 10^17^ ion/cm^2^, comparable to similar effects observed in ODS steels, indicates that the use of SiC ceramics can be carried out at higher temperatures (up to 1000 K), while an increase in temperature in ODS steels leads to accelerated swelling. At the same time, it should be noted that SiC ceramics also have a higher resistance to swelling, in contrast to nitride ceramics (AlN) [40], for which swelling was observed at fluences of 3–5 × 10^17^ ion/cm^2^ at room temperature (273 K), while for SiC ceramics, swelling was observed at similar fluences but when irradiated at temperatures of 700 K. It should also be noted that the transition from polycrystalline ceramics to 4H-SiC single crystals leads to a decrease in the resistance to swelling [41], which may be due to the fact that in polycrystalline ceramics, an increase in the swelling resistance occurs due to texture effects and different grain orientations that lead to the creation of additional boundary obstacles for the coalescence of helium bubbles. At the same time, tungsten is the most resistant to helium embrittlement and the formation of gas-filled bubbles, for which, as shown in the work [42], swelling is observed at a fluence of 7 × 10^17^ ion/cm^2^. At the same time, the authors of the work [42] showed that swelling and the formation of gas-filled bubbles lead not only to surface degradation but also to the propagation of deformation distortions associated with swelling during helium agglomeration deep into the sample, exceeding the maximum ion path length. These results confirm the observed decrease in hardness exceeding the He^2+^ ion path in ceramics at high irradiation fluences (above 5 × 10^17^ ions/cm^2^).

## 4. Conclusions

This paper presents the results of study of the influence of the temperature of irradiation with He^2+^ ions on the changes in the strength and thermophysical parameters of SiC ceramics depending on the irradiation fluence. The results obtained made it possible to determine the critical fluences in the case of variation in the irradiation temperature at which swelling of the near-surface layer occurs, which is expressed in the formation of gas-filled bubbles on the ceramic surface. In the case of irradiation of ceramics at a temperature of 700 K, the generation of gas-filled bubbles occurs at a fluence of 5 × 10^17^ ion/cm^2^, while at a temperature of 1000 K, the formation of bubbles is observed at a fluence of 10^17^ ion/cm^2^. Such a difference in the processes of bubble formation with temperature is due to the increased rate of helium migration at high temperatures, which leads to its agglomeration and the subsequent degradation of the surface. During this study, it was found that the most pronounced changes in strength characteristics (hardness, resistance to cracking and fracture) occur at fluences that are characterized by the generation of gas-filled bubbles in the damaged near-surface layer. At the same time, the analysis of changes in thermophysical properties and comparison of these changes with changes in strength characteristics showed a direct relationship between the deterioration in the strength properties of ceramics caused by the formation of gas-filled bubbles in the damaged layer with an increase in heat losses and a decrease in the thermal conductivity of ceramics, which can lead to destabilization of the core or fuel elements during long-term operation.

The obtained assessment results of the radiation resistance of carbide ceramics as a result of the accumulation of implanted helium in the near-surface layer can later be used when choosing materials for the core of new generation reactors. At the same time, the results of changes in mechanical strength during radiation damage accumulation can be used to determine the critical parameters of the core of nuclear reactors as well as operating modes at elevated temperatures and high doses of radiation.

## Data Availability

Not applicable.
